# A Functional Polymorphism C-509T in *TGFβ-1* Promoter Contributes to Susceptibility and Prognosis of Lone Atrial Fibrillation in Chinese Population

**DOI:** 10.1371/journal.pone.0112912

**Published:** 2014-11-17

**Authors:** Hailong Cao, Qing Zhou, Rongfang Lan, Oluf Dimitri Røe, Xin Chen, Yijiang Chen, Dongjin Wang

**Affiliations:** 1 Department of Thoracic and Cardiovascular Surgery, the Affiliated Drum Tower Hospital of Nanjing University Medical School, Nanjing, China; 2 Department of Cardiology, the Affiliated Drum Tower Hospital of Nanjing University Medical School, Nanjing, China; 3 Department of Thoracic and Cardiovascular Surgery, the First Affiliated Hospital of Nanjing Medical University, Nanjing, China; 4 Department of Cancer Research and Molecular Medicine, Norwegian University of Science and Technology (NTNU), Trondheim, Norway; National University of Singapore, Singapore

## Abstract

Transforming growth factor-β1 (*TGF-β1*) is an important mediator of atrial fibrosis and atrial fibrillation (AF). But the involved genetic mechanism is unknown. Herein, the *TGF-β1* C-509T polymorphism (rs1800469) was genotyped in a case-control study of 840 patients and 845 controls in Chinese population to explore the association between the polymorphism and susceptibility and prognosis of lone AF. As a result, the CT and/or TT genotypes had an increased lone AF risk [adjusted odds ratio (OR) = 1.50 for CT, OR = 3.72 for TT, and OR = 2.15 for CT/TT], compared with the *TGF-β1*CC genotype. Moreover, patients carrying CT/TT genotypes showed a higher possibility of AF recurrence after catheter ablation, compared with patients carrying CC genotype. In a genotype-phenotype correlation analysis using 24 normal left atrial appendage samples, increasing gradients of atrial *TGF-β1* expression levels positively correlated with atrial collagen volume fraction were identified in samples with CC, CT and TT genotypes. The *in vitro* luciferase assays also showed a higher luciferase activity of the -509T allele than that of the -509C allele. In conclusion, the *TGF-β1* C-509T polymorphism is involved in the etiology of lone AF and thus may be a marker for genetic susceptibility to lone AF and predicting prognosis after catheter ablation in Chinese populations. Therefore, we provide new information about treatment strategies and our understanding of *TGF-β1* in AF.

## Introduction

Atrial fibrillation (AF), the most common cardiac arrhythmia, is associated with increased morbidity, mortality and cost [1]. To date, the precise mechanism of AF remains largely unknown. It is particularly true for patients with lone AF (LAF), which is AF in the absence of heart disease or comorbidities predisposing to the arrhythmia. However, increasing evidence showed that the atrial fibrosis is essential for the development and progression of AF [2]–[3].

It is accepted that transforming growth factor-β1 (*TGF-β1*) is the most powerful profibrotic factor, stimulating the secretion of collagen into the extracellular matrix by fibroblasts [4]. Over-expression of *TGF-β1* selectively induced atrial fibrosis, leading to increased conduction heterogeneity and AF vulnerability without affecting the cellular electrophysiology [5]. Inhibition of *TGF-β1* expression by Tranilast significantly reduced the atrial fibrosis, as a result, reduced conduction abnormalities and AF vulnerability were observed [6]. These studies suggest that *TGF-β1* plays an essential role in inducing AF.

In the human *TGF-β1* gene, three polymorphisms in the promoter are presently known [7]. It was proposed in a previous *in vitro* study that the differences in the induced expression and production of *TGF-β1* were a consequence of genetic sequence polymorphisms in the regulatory promoter region [8]. Nevertheless, in Chinese and other Asians, the genetic polymorphism in the promoter was detected only in -509 [9]–[10]. Therefore, we hypothesized that the *TGF-β1* C-509T polymorphism was associated with LAF risk and AF recurrence after catheter ablation in Chinese population. Furthermore, we also designed to explore whether this polymorphism had an effect on *TGF-β1* expression *in vitro* and left atrial appendage (LAA) tissue.

## Materials and Methods

### 2.1. Ethics statement

This hospital-based case-control study was approved by the Review Boards of the Affiliated Drum Tower Hospital of Nanjing University Medical School and the First Affiliated Hospital of Nanjing Medical University (2007-NJEA-10). All subjects provided written informed consent to be included in the study. The study was conducted according to the Helsinki Declaration and approved by the ethics committees of the respective institutions (2007-NJEA-10). We have complied with the World Medical Association Declaration of Helsinki regarding ethical conduct of research involving human subjects.

### 2.2. Study Subjects

We consecutively recruited 2786 non-valvular AF patients between October 2007 and June 2013. Each patient donated 5 ml venous blood upon admission to the hospital and was interviewed to collect demographic data and clinical information. Among them, a total of 860 patients were diagnosed as lone AF, which was based on the following criteria [11]: age at first diagnosis of AF <60 years, no past cardiovascular history, no evidence suggesting ischemic heart disease, no cardiomyopathy, no heart failure, no valvular heart disease, no diabetes, no hypertension within two years of the onset of AF, no hyperthyroidism. Sex- and age-matched 860 AF-free control subjects with written informed consent were genetically unrelated to the cases. They were recruited from healthy subjects without individual history of AF and other chronic diseases. All subjects were genetically unrelated ethnic Han Chinese.

Among all the subjects, genotyping was failed in 20 cases (2.59%) and 15 controls (1.88%) due to DNA quality or quantity. Therefore, 840 lone AF cases and 845 healthy controls were finally included in the susceptibility analyses. Among the 840 cases, 725 received first-time catheter ablation and were successfully cardioverted to stable SR. During the follow-up, 112 patients were lost and 3 cases died because trauma or cerebrovascular accident. Therefore, a total of 610 patients had complete follow-ups and clinical information.

### 2.3. Follow-Up

AF-free time was calculated from the date of ablation to the date of recurrence or last follow-up. Atrial arrhythmias that occurred during the first 2 months after ablation, which is considered a blanking period [12], were not counted as recurrences. Antiarrhythmic medications, including amiodarone, metoprolol or propafenone, were generally continued to the end of third month after ablation unless recurrent arrhythmia indicated the need for continued treatment. All patients with documented arrhythmia and those maintained on antiarrhythmics for control of AF beyond the blanking period were counted as recurrences.

Patients had scheduled clinical visits, 12-lead ECG, and 24-hour Holter monitoring at 3, 6, and 12 months after ablation and then yearly after the first year. Moreover, patients would receive ECG monitoring in local clinics at anytime if they had AF-related symptoms. AF recurrence was identified by symptoms with ECG documentation of an atrial tachyarrhythmia lasting ≥30 seconds on a 12-lead ECG or Holter monitor recording. AF recurrence during the follow-up was considered censored.

### 2.4. Genotyping

Genomic DNA was isolated from leukocytes of venous blood by proteinase K digestion and phenol/chloroform extraction. The *TGF-β1* C-509T polymorphism was determined using the PCR–restriction fragment length polymorphism method. The PCR primers for the *TGF-β1* C-509T polymorphism were 5′-GCTAAGGCATGGCACCGCTT-3′ (forward) and 5′-GAAGGAGGGTCTGTCAACATGGG-3′ (reverse). PCR was performed in a total volume of 20 mL containing 50 ng genomic DNA, 10×Taq buffer, 0.02 mmol⋅L^−1^ of MgCl_2_, 0.05 mmol⋅L^−1^ of dNTP mix, 10 pmol⋅mL^−1^ of each primer and 1 U Taq DNA polymerase. After initial denaturation at 95°C for 5 minutes, the reaction was carried out at 95°C denaturation for 30 seconds, annealing for 40 seconds at 62°C and extension for 45 seconds at 72°C for a total of 34 cycles, and a final elongation at 72°C for 10 minutes. The 270-bp PCR products were digested by the restriction enzyme (*Eco81I*, SauI) (MBI Fermentas, Vilnius, Lithuania) at 37°C overnight. The digested products were then analyzed by electrophoresis in 3% agarose gel stained with 0.5% ethidium bromide and photographed under UV illumination. The C allele was cut into 198-bp and 72-bp fragments, whereas the T allele was not digested. The polymorphism analysis was carried out by two persons independently in a blinded manner. More than 15% of the samples were randomly selected for confirmation, and the results were 100% concordant.

### 2.5. LAA samples

In order to determine the expression of *TGF-β1* and interstitial fibrosis, we collected 24 LAA tissues from healthy heart donors for transplantation. They were trauma victims and were free of cardiovascular pathology and documented AF. LAA specimens were obtained before perfusion. A part of each LAA was fixed in paraformaldehyde for histology, and the others were immediately snap-frozen in liquid nitrogen for laboratory analysis.

### 2.6. Real-time quantitative RT-PCR

The primers of *TGF-β1* (Forward primer [F]: 5′-CTAATGGTGGAAACCCACAACG-3′, Reverse primer [R]: 5′-TATCGCCAGGAATTGTTGCTG-3′, NM_000660) and glyceraldehydes 3-phosphate dehydrogenase (GAPDH) (Forward primer [F]: 5′-ATGGGGAAGGTGAAGGTCG-3′, Reverse primer [R]: 5′-GGGGTCATTGATGGCAACAATA-3′, NM_002046) were synthesized by Invitrogen Co., Hong Kong, China. Total RNA was isolated from frozen RAAs by acid-phenol extraction in the presence of chaotropic salts (TRIzol, Invitrogen) and subsequent isopropanol ethanol precipitation. The synthesis of cDNA was according to the manufacturer’s instructions with the reverse transcriptase kit (Promega Co., US). The real-time PCR was performed using the LightCycler 480 system (Roche Diagnostics, Switzerland) with a total volume of 20 µl containing 10 µl 2×Master Mix SYBR Green I (Takara, Japan), 0.25 µM forward primers, 0.25 µM reverse primers, 2 µl cDNA template, and H_2_O to a final volume of 20 µl. The protocol of real-time PCR consisted of 40 cycles, and cycling parameters were as follows: denaturation at 94°C for 10 seconds, annealing at 61°C for 15 seconds and extension at 72°C for 20 seconds. The results were analyzed using Roche LightCycler 480 software. Data of transcripts were calculated relative to GAPDH using the 2^−ΔΔCt^ method. The measurements of each sample were performed in triplicate.

### 2.7. Western blot

Frozen LAAs were used for protein isolation as described previously [13]. Proteins (40 µg/lane) were separated by sodium dodecyl sulfate polyacrylamide gel electropheresis and transferred onto Polylinylidene Fluoride membranes using a Bio-Rad semidry transfer system (Bio-Rad). The membranes were blocked with 5% non-fat dry milk and then probed with mouse monoclonal *TGF-β1* (ab27969, Abcam, USA) and horseradish peroxidase (HRP)-conjugated mouse monoclonal anti-GAPDH (KC-5G5, KangChen Biotech, China). The working dilutions were 1∶2000 (*TGF-β1*) and 1∶5000 (GAPDH). The resulting reaction was visualized using HRP-conjugated anti-mouse secondary antibody (Santa-Cruz Biotechnology, the Netherlands), followed by incubation with ECL Western Blot Detection Kit (Amersham, the Netherlands) for 1 minute. The blots were exposed to Kodak film for 5 minutes and immunoreactive bands developed for quantification using The Discovery Series image analysis software (Bio-Rad) normalized by the corresponding value of GAPDH. Experiments were repeated three times and the mean was scored.

### 2.8. Masson’s trichrome staining

After fixation with 4% paraformaldehyde in phosphate-buffered saline (PH: 7.4) for 24 h, the tissues were subjected to alcoholic dehydration and embedded in paraffin. 4 µm serial sections were sliced and subjected to Masson’s trichrome staining to highlight collagen fibers. Collagen volume fraction (CVF) was determined by the HPISA 100 chromatic color pathological analysis system (Olympus, Japan) using five random images from each slide and five slides per sample, and the mean values of CVF were obtained by one investigator blinded to the groups.

### 2.9. Promoter functional assay

The isolation and culture of mouse atrial fibroblasts were previously described [14]. The *TGF-β1* promoter-luciferase reporter plasmids containing either -509C or -509T sequences were prepared by amplifying the 270-bp *TGF-β1* promoter region by using primers with restriction sites. The primers were 5′-GCTAAGGCATGGCACCGCTT-3′ (forward) and 5′-GAAGGAGGGTCTGTCAACATGGG-3′ (reverse), including the *KpnI* and *XhoI* restriction sites. The amplified fragments were then sequenced to confirm that there were no errors in matched nucleotides and the plasmid encompassed either -509C or -509T allele. The amplified fragments and luciferase reporter vectors (pGL3)-basic vector (Promega, Madison, WI, USA) were cleaved by using the *KpnI* and *XhoI* enzymes (Promega, USA), and the fragments were then cloned into the pGL3-basic vector. After cloning, the vectors were sequenced to confirm the orientation and integrity of the inserts of each construct. For transfections, mouse atrial fibroblasts were seeded onto 24-well plates (100,000 cells per well), and each well was transfected with 1 µg of the vector DNA with either -509C or -509T allele, using Lipofectamine 2000 (Invitrogen, Carlsbad, CA, USA). As an internal standard, all plasmids were cotransfected with 8 ng pRL-SV40, which contained the Renilla luciferase gene. The pGL3-basic vector without an insert was used as a negative control. After 48 hours of incubation, cells were collected and analyzed for luciferase activity with the Dual-Luciferase Reporter Assay System (Promega, Madison, WI, USA).

### 2.10. Statistical analysis

Differences in the distributions of demographic characteristics, clinical variables, and frequencies of genotypes of *TGF-β1* C-509T polymorphism between the cases and controls were evaluated using the Student’s t-test (for continuous variables) and *χ^2^* test (for categorical variables). Hardy–Weinberg equilibrium was tested using a goodness-of-fit χ^2^ test. The association between the *TGF-β1* C-509T polymorphism and AF risk was estimated by computing odds ratios (ORs) and their 95% confidential intervals (CIs) from multivariate logistic model. An allele-specific difference in luciferase activity was also tested using the Student’s t-test. For the comparison of atrial expression of *TGF-β1* and the degree of atrial fibrosis, one-way ANOVA test (normally distributed) or Mann-Whitney test (2 groups, non-normally distributed) and Kruskal-Wallis test (n groups, non-normally distributed) were used among the three genotypes. Spearman correlation analysis was applied to assess the association between expression of *TGF-β1* and CVF in LAAs. The Kaplan-Meier method, log-rank test, and Cox survival regression model were used to determine factors predictive of AF outcome after ablation. *p*<0.05 was considered statistically significant, and all statistical tests were two-sided. All the statistical analyses were performed with Statistical Analysis System software (v.9.1.3; SAS Institute, Cary, NC, USA).

## Results

### 3.1. Characteristics of the study population

The characteristics of LAF group and Control group enrolled in this study were shown in Table 1. There were no significant differences in the distribution of the age, sex, body mass index (BMI), cigarette smoking, alcohol intake and hypercholesterolaemia. Left atrial dimension (LAD) was significantly lager in LAF than control. In LAF group, age at first diagnosis of AF was 45.6±11.3 years, and 70.5% was paroxysmal AF. Medications before enrollment in LAF group were Digitalis (n = 90), Propafenone (n = 135), Amiodarone (n = 155), Beta-blocker (n = 258), angiotensin-converting enzyme inhibitor/angiotensin receptor blocker (n = 171), Calcium-channel blocker (n = 50), Warfarin (n = 311) and Statins (n = 20).

**Table 1:** Baseline characteristics of subjects with LAF and controls.

Variables	LAFgroup	Control group	*p*-value
Patient number *(n)*	840	845	-
Sex, M/F (*n*)	561/279	562/283	0.904
Age at enrollment (yrs)	52.8±10.9	52.8±14.9	0.938
Age at first diagnosis of AF (yrs)	45.6±11.3	N/A	-
Paroxysmal/Persistent AF (*n*)	592/248	N/A	-
Body mass index (kg/m^2^)	23.8±2.9	23.9±3.0	0.501
Cigarette smoking (*n*)	138	168	0.066
Alcohol intake > = 1 drink per day (*n*)	98	96	0.844
Hypercholesterolaemia (*n*)	46	65	0.067
Left atrial dimension (mm)	39.1±6.7	31.1±3.6	<0.001
Medications before enrollment (*n*)			
Digitalis	90	N/A	-
Propafenone	135	N/A	-
Amiodarone	155	N/A	-
Beta-blocker	258	N/A	-
ACE-I/ARB	171	N/A	-
Calcium-channel blocker	50	N/A	-
Warfarin	311	N/A	-
Statins	20	N/A	-

Values are presented as mean±SD or number of patients.

ACE-I, angiotensin-converting enzyme inhibitor; ARB, angiotensin receptor blocker.

### 3.2. TGF-β1 C-509T polymorphism and LAF risk

The genotype and allele distributions of the *TGF-β1* C-509T polymorphism in the cases and controls are shown in Table 2. The observed genotype frequencies for this polymorphism were in Hardy–Weinberg equilibrium in the controls (*χ^2^* = 0.286, *p* = 0.593). The frequencies of the CC, CT and TT genotypes were 18.1%, 40.2% and 41.7%, respectively, among the cases, and 32.3%, 47.8% and 19.9%, respectively, among the controls. After adjusting for possible confounders (age, sex, body mass index, smoking status, drinking status, and hypercholesteremia), subjects carrying CT or TT or CT/TT genotypes had an increased risk of LAF (adjusted OR = 1.50(1.17–1.92) for CT, 3.72 (2.83–4.88) for TT and 2.15 (1.71–2.70) for CT/TT; *p*_trend_<0.001), compared with CC homozygote, and the frequency of the T allele being higher than C allele among LAF subjects (*p*<0.001).

**Table 2:** Genotype and allele frequencies of the *TGF-β1* C-509T polymorphism between the cases and controls and their associations with risk of LAF.

Genotypes	LAF (*n* = 840)		Controls (*n* = 845)		*p* a		Adjusted OR(95% CI)^b^
	*n*	%	*n*	%		(95% CI)^b^	
CC	152	18.1	273	32.3		1.00 (reference)	
CT	338	40.2	404	47.8	0.001	1.50 (1.17–1.92)	
TT	350	41.7	168	19.9	<0.001	3.72 (2.83–4.88)	
CT/TT	688	81.9	572	67.7	<0.001	2.15 (1.71–2.70)	
C allele	642	38.2	950	56.2	<0.001		
T allele	1038	61.8	740	43.8			
*p*_trend_					<0.001		

a Two-sided χ^2^ test for either genotype distributions or allele frequencies between the cases and controls.

b Adjusted for age, sex, body mass index, smoking status, drinking status, and hypercholesteremia in logistic regression model.

### 3.3. Factors associated with AF recurrence after ablation

**Figure 1:**
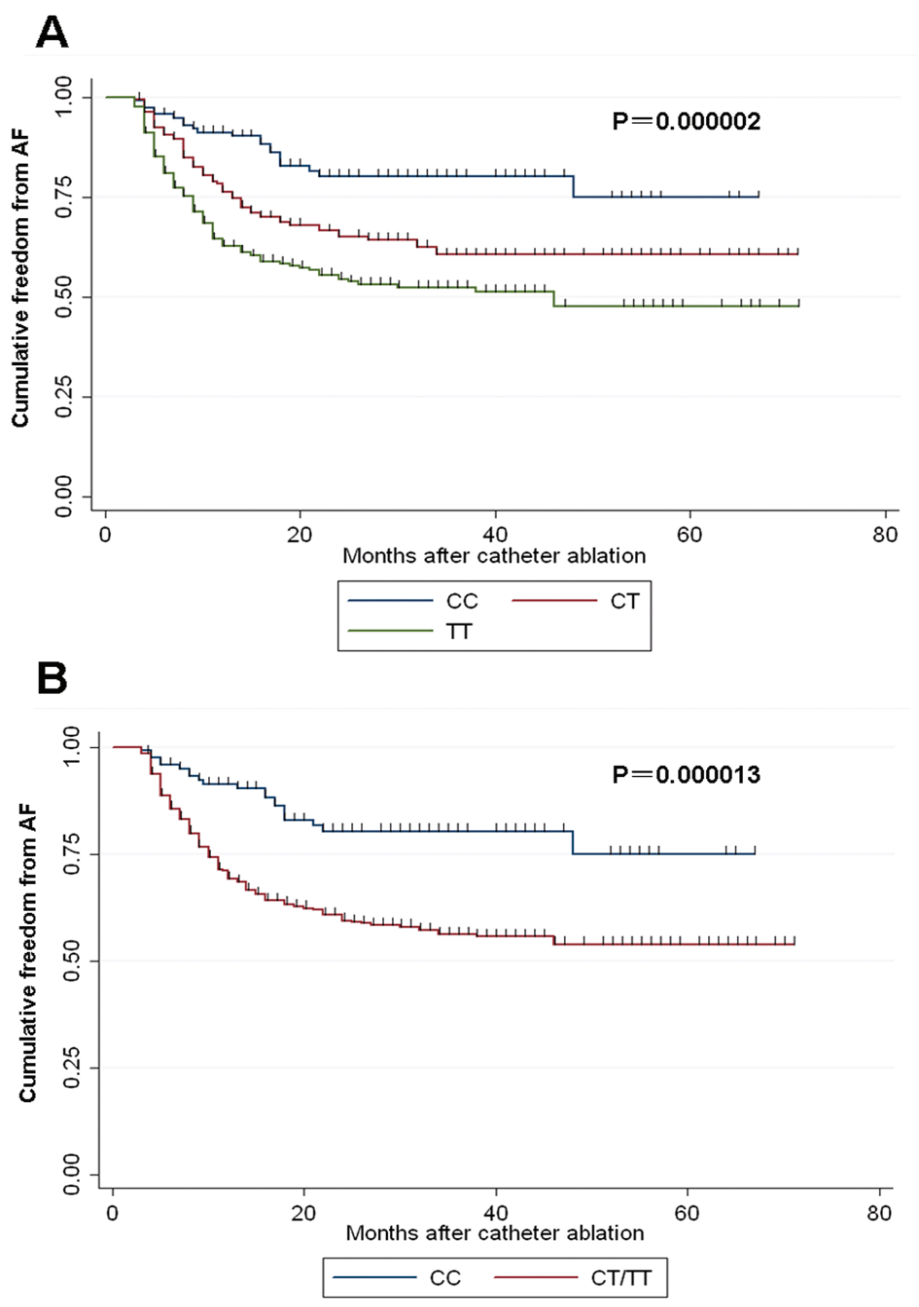
Kaplan-Meier survival curves showing freedom from AF recurrence after catheter ablation according to *TGF-β1* C-509T polymorphism. (A) Survival free from AF recurrence in CC (n = 120), CT (n = 231) and TT (n = 259) groups. (B) Survival free from AF recurrence in CC (n = 120) and CT/TT (n = 490) groups.

Over a median follow-up of 17.5 months (range, 3.5 to n 71 months) after ablation, 207 patients (33.9%) had AF recurrence and 403 patients (66.1%) remained in SR. I multivariate Cox proportional hazards analysis, factors associated with arrhythmia recurrence were found to be persistent AF (versus paroxysmal AF; HR = 4.289 (3.217–5.718); *p*<0.001), larger left atrial dimension (HR = 5.212 (3.937–6.900); *p*<0.001), CT/TT genotype (versus CC genotype; HR = 4.121 (2.597–6.539); *p*<0.001) (Table 3). In addition, Kaplan-Meier survival estimates showed that LAF patients carrying different *TGF-β1* C-509T genotypes had different proportion of AF recurrence (*p*<0.001) (Figure 1A). Moreover, CT/TT genotypes also had a higher proportion of AF recurrence after ablation, compared with CC genotype (*p*<0.001) (Figure 1B).

**Table 3:** Results of Cox multivariate regression analysis on cumulative AF recurrence after catheter ablation.

Final variables	β	SEM	HR	95% CI	*p*-value
Hypertension at enrollment	0.289	0.159	1.335	0.977–1.824	0.070
Persistent AF	1.456	0.147	4.289	3.217–5.718	<0.001
Postoperative Statins	–0.211	0.145	0.810	0.610–1.076	0.146
Left atrial dimension	1.651	0.143	5.212	3.937–6.900	<0.001
C-509T (*CT/TT* vs. *CC*)	1.416	0.236	4.121	2.597–6.539	<0.001

β, regression coefficient; HR, hazard ratio.

### 3.4. TGF-β1 C-509T polymorphism and luciferase activity

**Figure 2:**
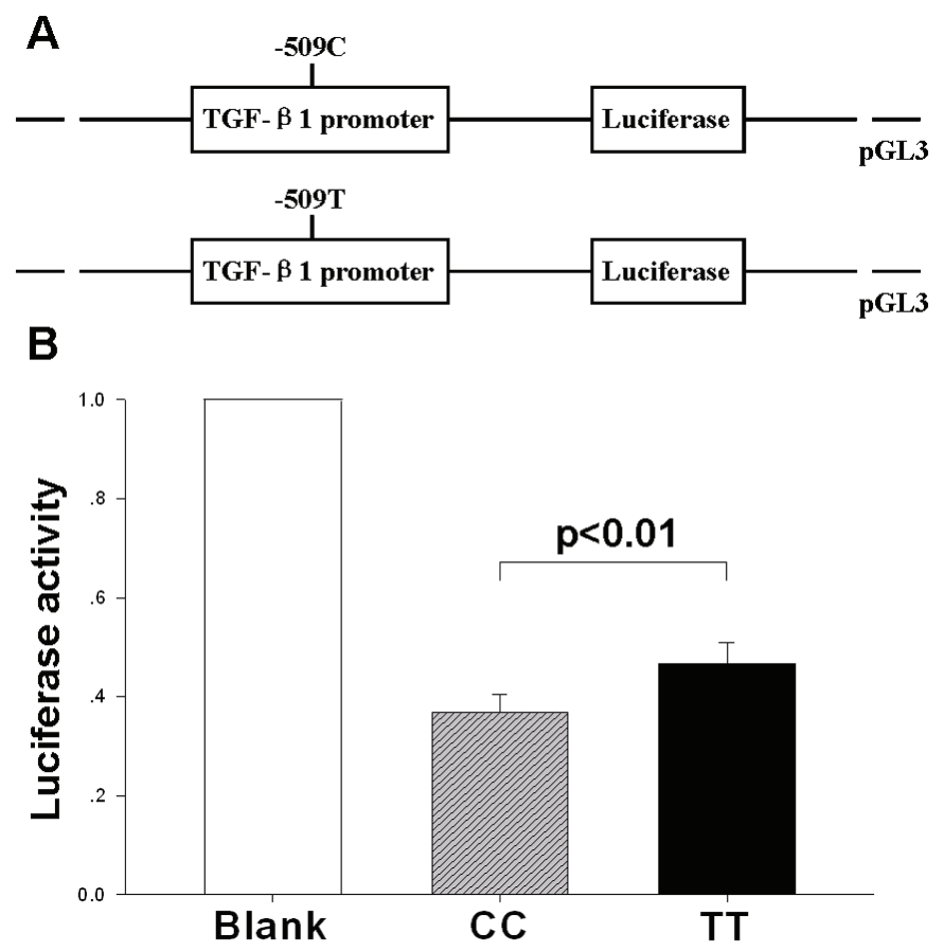
Effect of the C-509 T polymorphism in the *TGF-β1* promoter activity. (A) Schematic representation of reporter plasmids containing the -509C or -509 T allele, which was inserted upstream of the luciferase reporter gene in the pGL3 basic plasmid. (B) Two constructs were transiently transfected into the mouse cardiac fibroblasts. The luciferase activity of each construct was normalized against the internal control of Renilla luciferase (blank). Values are mean±SD.

As shown in Figure 2, the vector with the -509T allele had an increase in the relative luciferase activity, compared with that with the -509C allele (*p*<0.01). These results suggested that the -509T allele may lead to a higher expression levels of *TGF-β1* mRNA than the -509C allele.

### 3.5. Expression of TGF-β1 and the degree of atrial fibrosis among genotypes

**Figure 3:**
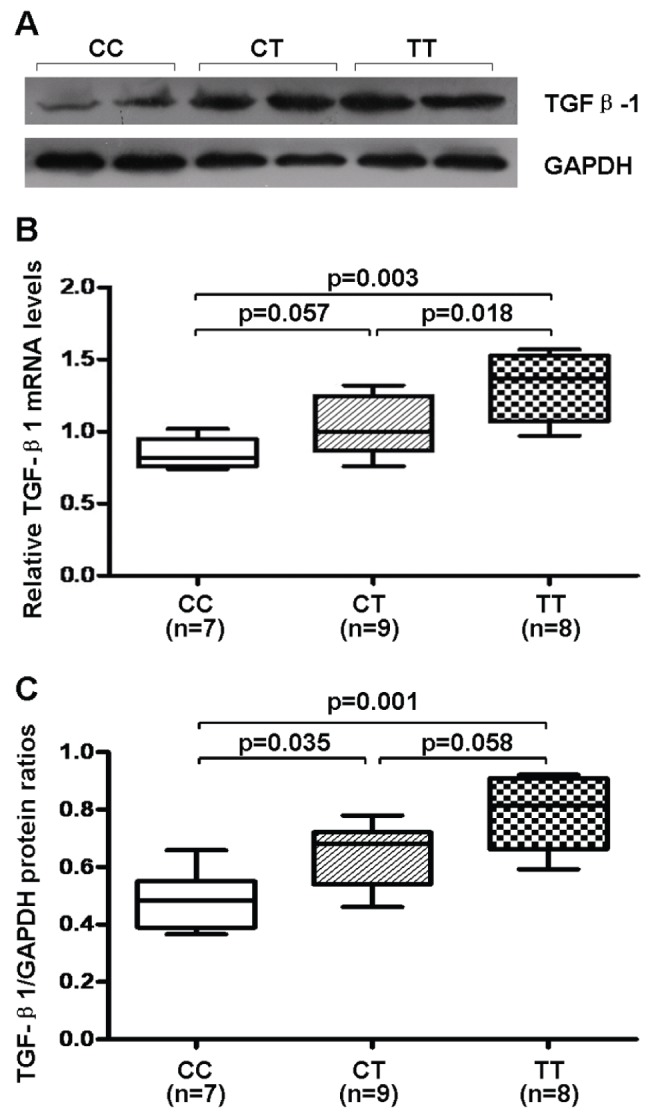
Atrial expression of *TGF-β1* among different genotypes in healthy heart donors. (A) Representative Western immunoblots. (B) Gene expression in left atrial appendages (LAAs). (C) Semi-quantitative protein content in LAAs. Boxes show interquartile ranges, and bars represent the 10th and 90th percentiles.

In the 24 LAA specimens, 7 were of the CC genotype, 9 of the CT genotype, and 8 of the TT genotype. As shown in Figure 3B, Real-time quantitative RT-PCR assay showed an increasing gradient of gene expression of *TGF-β1* in the groups carrying CC, CT and TT genotypes, although there was merely a borderline significant difference between CC and CT groups (*p* = 0.057).

Western blot analysis (Figure 3A, C) showed an increased expression of *TGF-β1* in CT and TT groups than CC group, whereas the difference was borderline between CT and TT groups (*p* = 0.058).

**Figure 4:**
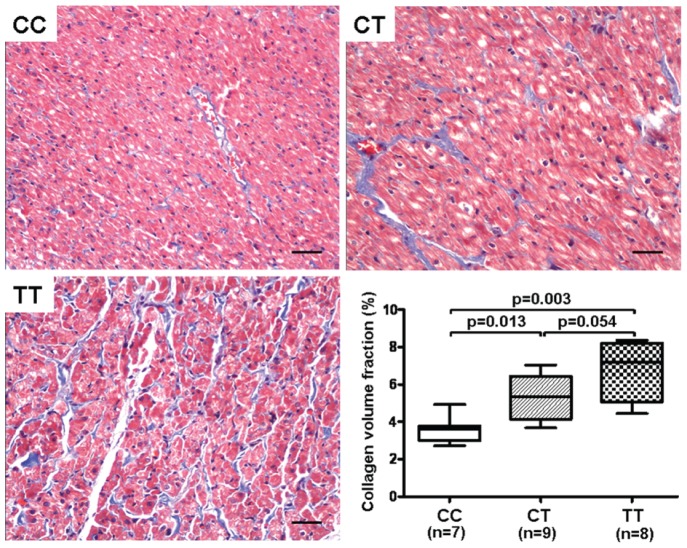
Representative photomicrographs of Masson staining showing the interstitial collagen (stained blue) (×200, bar = 50 µm). Collagen volume fraction is used to evaluate the degree of fibrosis.

Interstitial collagen (stained blue), revealed by Masson staining and expressed as CVF, was lowest in CC group, followed by CT and TT groups (Figure 4). However, there was merely a borderline significant difference between CT and TT groups (*p* = 0.054).

**Figure 5:**
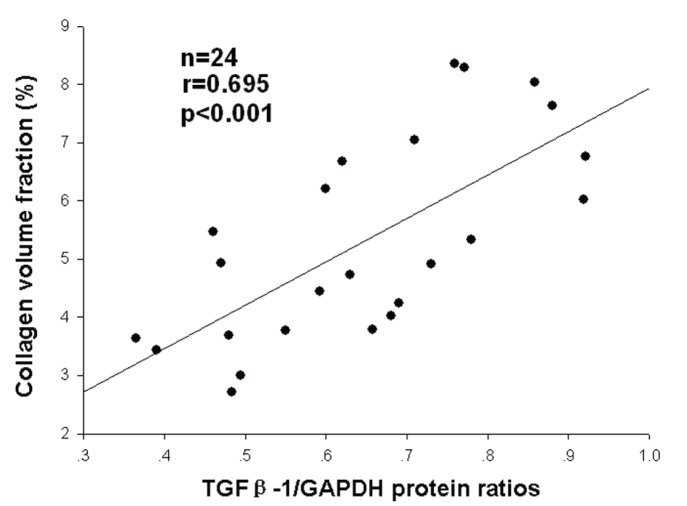
Correlation between atrial protein expression of *TGF-β1* and collagen volume fraction in LAAs.

The correlation test indicated a strong positive correlation between atrial protein expression of *TGF-β1* and CVF in LAAs (r = 0.695, p<0.001; Figure 5).

## Discussion

In this case-control study, we investigated the relationships between *TGF-β1* C-509T polymorphism and susceptibility and prognosis of LAF in Chinese population. We made several new findings. First, our results revealed that the CT and/or TT genotypes had an increased LAF risk [adjusted OR = 1.50 for CT, OR = 3.72 for TT, and OR = 2.15 for CT/TT], compared with the *TGF-β1*CC genotype. Second, we demonstrated that LAF patients carrying CT/TT genotypes had a higher possibility of AF recurrence after ablation, compared with patients carrying CC genotype. Furthermore, we validated both in vitro and vivo that T allele had higher expression of *TGF-β1* and more aggravated atrial interstitial fibrosis than C allele. However, a recent study determining the same polymorphism in also Chinese Han population found no association between the rs1800469 polymorphism and the risk of AF under the dominant, recessive and additive genetic models [15]. It could be due to their enrolled patients who had kinds of underlying disease (hypertension, coronary heart disease and so on) and were much older. But herein we only recruited LAF cases who were probably determined by genetics. Moreover, they had much fewer cases. So the inherent selection bias could not be completely excluded. To the best of our knowledge, this is the first study to investigate the potential functional polymorphism C-509T of *TGFβ-1* promoter in susceptibility and prognosis of AF.

Currently, atrial remodeling has been demonstrated as the most important pathophysiological mechanism of AF. Electrical remodeling is reversible, but in patients with a long history of AF or severe comorbidities, structural remodeling has become severe and irreversible, causing progression of AF and making it challenging to restore and maintain SR [16]. Among atrial structural remodeling including myolysis, apoptosis, fibrosis and inflammation, atrial interstitial fibrosis is the most important process in development and progression of LAF [16]. *TGF-β1* is a cytokine that modulates the tissue fibrosis [4]. Recent studies showed that over-expression of *TGF-β1* as well as an increase of atrial fibrosis was observed in atrial specimens from patients with AF [17]–[19]. High-expression of atrial *TGF-β1* indicated a higher recurrence rate and a poor recovery of atrial mechanical contraction after surgical maze procedure [20]–[21]. It was speculated and further confirmed in an transgenic animal model that atrial over-expression of *TGF-β1* selectively induced atrial interstitial fibrosis, contributing to AF vulnerability [5], [19]. Inhibition of *TGF-β1* expression by an antiallergic drug named Tranilast decreased the atrial fibrosis and AF vulnerability [22]. These studies suggest that the *TGF-β1* attributes to development and recurrence of AF via triggering atrial fibrosis.

The human *TGF-β1* gene is located on chromosome 19q13 and can be transcribed and translated to form a 390 amino acid propeptide. The C-509T polymorphism of *TGF-β1* gene is located in the promoter region which is relative to the first major transcription start site and was found to be related to transcriptional activity and plasma concentration of *TGF-β1* [23]. A recent study firstly investigated the polymorphisms of *TGF-β1* T +869C at codon 10 and G +915C at codon 25 in susceptibility of AF in essential hypertensive subjects, and found the latter was associated with occurrence of AF and serum *TGF-β1* level in this population [24]. In this study, our results suggested that the -509T allele leaded to a higher expression level of *TGF-β1* both *in vivo* and *in vitro* correlated with more severe interstitial fibrosis. Therefore, it is suggested that increased *TGF-β1* expression by -509T allele may induce overproduction of extracellular matrix components such as collagen by myofibroblasts, resulting in progressive atrial augmentation, fibrosis, and probably the susceptibility and recurrence of LAF. It is further speculated that *TGF-β1* gene polymorphisms could regulate expression of *TGF-β1* and play a role in the development and prognosis of LAF.

According to recent guidelines, catheter ablation is considered as initial therapy in selected patients, especially in those with symptomatic paroxysmal AF with no or minimal heart disease and those failed to antiarrhythmic drug therapy [25]. So it is very important to evaluate the prognosis of LAF after catheter ablation. The previous studies demonstrated that bigger left atrial dimension and persistent AF significantly increased the risk of recurrent after ablation [26]–[28], which was consistent with our findings. Interestingly, we found that *TGF-β1* C-509T polymorphism could also be an independent predictor of recurrence after AF ablation in LAF patients. Patients carrying *CT/TT* genotypes may have higher atrial expression of *TGF-β1* as well as more severe atrial interstitial fibrosis, therefore may be not the suitable candidate for isolated catheter ablation.

### 4.1. Clinical perspectives

Atrial structural remodeling, particularly interstitial fibrosis, limits the efficacy of existing therapies for AF in clinic. Accordingly, attenuation of structural remodeling, so-called upstream therapy, has increasingly become the focus of attention. It can prevent both the development of AF (primary prevention) and AF recurrence after cardioversion (secondary prevention), therefore becomes a promising approach for AF treatment [29]. Previously, it had been demonstrated that *TGF-β1* neutralization via polyclonal antibodies could result in the down-regulation of extracellular matrix gene expression in rats [30]. Likewise, regulating excessive expression of endogenic *TGF-β1* may also help to prevent fibrosis in atria. Taken together, we propose that inhibiting excessive expression of endogenic *TGF-β1* by targeting the *TGF-β1* C-509T polymorphism can be a new promising upstream therapy of AF. Additionally, profile evaluation of *TGF-β1* gene polymorphisms has the potential to be used clinically as a routine pre-ablation assessment, and together with other factors including AF type and left atrial diameter, may provide a more integrated picture for physicians to evaluate the clinical status of LAF patients. Paroxysmal AF patients carrying CC genotype of *TGF-β1* C-509T polymorphism without significantly enlarged left atria may be the optimal candidates for catheter ablation.

### 4.2. Limitations

Firstly, although 24 LAA specimens were statistically large enough to support our findings, the small sample size indeed limited the statistical power. Secondly, despite regular screening of patients with outpatient visits and ambulatory ECG monitoring, the detection of all episodes of AF recurrence, particularly asymptomatic ones, is very difficult to establish. Therefore, we may have underestimated the true incidence of AF recurrence in our study. Finally, although we applied a rigorous epidemiological design in selecting study subjects and adjusted for confounding factors in further statistical analysis to minimize the potential biases, inherent selection bias cannot be completely excluded.

## Conclusion

Our study showed that the *TGF-β1* C-509T polymorphism was associated with LAF risk and AF recurrence after catheter ablation by affecting the expression level of *TGF-β1* as well as the degree of atrial interstitial fibrosis. These findings enhanced our knowledge of the role of *TGF-β1* in AF, and suggested that the C-509T polymorphism could be a functional genetic target for developing new treatment strategies and guide the physicians for catheter ablation as a useful marker.

## Supporting Information

Data S1 Original data of demographic characteristics for LAF and SR groups.
(XLS)

Data S2 Original data of in vivo and in vitro functional data.
(XLS)

Data S3 Original data of Follow-Up data.
(XLS)

Ethics S4 Ethic certification for this study.
(PDF)
