# Supplementary material for: A Functional Polymorphism C-509T in TGFβ-1 Promoter Contributes to Susceptibility and Prognosis of Lone Atrial Fibrillation in Chinese Population
Source: PLoS One. 2014 Nov 17;9(11):e112912. doi: 10.1371/journal.pone.0112912 (PMC4234495; doi:10.1371/journal.pone.0112912)
Supplement: Ethics S4 — Ethic certification for this study. (PDF) [file pone.0112912.s004.pdf]

南京大学医学院附属鼓楼医院医学临床研究伦理审查表

申请日期：2012 年 2 月 7 日

项目名称：TGF-β1 基因单核苷酸多态性在心房颤动发生和预后中的作用
项目负责人：曹海龙 职称：医师 单位：南京大学医学院附属鼓楼医院
项目联系人：曹海龙 电话：13675186233 信箱：shuqu_1982@sina.com
合作研究单位：南京医科大学第一附属医院 负责人：薛磊 电话：13851636605
请求审查类型：2012 年度国家自然科学基金申请项目
递交审查资料<br>☑实验方案 ☑知情同意书 □其他资料

涉及人的生物医学研究内容及研究方案摘要

心房颤动是临床上最常见的心律失常，发病率逐年增加。它可诱发和加重心力衰竭，增加血栓栓塞的发生率，已成为威胁公众健康的重要因素。房颤最重要的病理基础就是心房间质纤维化，而 TGF-β1 在其中起着重要作用。但由于其准确的调节机制尚不清，因而限制了其作为"上游治疗"在临床中的应用。本课题紧密结合临床，应用分子流行病学技术探讨 TGF-β1 基因多态性在非瓣膜病房颤的发生和消融后复发中的作用，并进一步在离体与在体功能学水平揭示其中精确的作用机制。为基于调节 TGF-β1 的房颤"上游治疗"奠定一定的理论基础和提供有效的干预靶点。此外，通过筛选包括 TGF-β1 基因多态性在内的临床指标用于术前判断行消融治疗患者的预后，从而进一步充实射频消融术的适应症，并为消融治疗术后其他相关辅助干预提供询证医学依据。

涉及人的生物医学研究内容：

1、运用病例-对照研究方法，探讨TGF-β1基因单核苷酸多态性与非瓣膜病房颤发生风险之间的关系，并通过单倍体频率分析和分层分析明确不同多态性位点之间和临床相关因素（如年龄、性别、体重指数、吸烟、饮酒、高血脂等）与相关多态性之间的交互作用和协同效应。

2、根据非瓣膜病房颤患者消融术后的随访资料，将TGF-β1基因多态性和其他可能影响预

后的相关指标行生存回归分析，明确相关多态性位点是否能够影响和预测房颤消融术后的复发，并同时筛选影响预后的临床指标。

3、对于影响房颤易感性的TGF-β1基因多态性位点，从在体水平探讨这些多态性对TGF-β1基因转录活性以及蛋白表达和心房间质纤维化程度的影响。采用real-time PCR方法分析不同基因型的左心耳组织中TGF-β1转录水平的差异；采用Western blot实验方法分析不同基因型的左心耳组织中TGF-β1蛋白表达水平的差异；采用特殊染色的方法分析不同基因型的左心耳组织中纤维化程度的差异。

涉及人的生物医学研究方案：

1、分子流行病学研究：

（1）研究对象：收集非瓣膜病房颤患者2000例及无房颤病史并年龄、性别匹配的健康对照2000例。所有房颤患者均为住院治疗病例，有完善的病历和辅助检查资料，出院前由统一培训的调查员完善所有临床资料的收集。对照组均为体检中心无房颤病史和心电图表现的健康人群。在签署书面知情同意后，收集每一个病例的血常规剩余血，当天离心分离血浆、白细胞和红细胞后-80°C冰箱冷冻保存备用。所有行射频消融的房颤患者术后由统一培训的调查员行长期的电话、门诊随访，直至有心电图证据的房颤复发。

（2）基因组DNA抽提：在解冻的白细胞中加入细胞裂解液，裂解离心后，在沉淀内加蛋白酶K消化，以酚/氯仿抽提DNA。DNA经鉴定合格后4 °C保存备用。

（3）基因型分析：采用PCR-RFLP和/或TaqMan检测方法分析SNPs的基因型在病例组和对照组人群中的频率分布。

（4）风险性分析：将每个TGF-β1基因多态性位点的不同基因型对房颤的易感性行危险度分析，并用逻辑回归分析对相关临床指标进行校正。单倍体频率分析这些多态性位点之间的交互作用。分层分析这些多态性位点与相关临床因素之间的协同效应。

（5）生存回归分析：将相关多态性位点与其他可能影响房颤消融术后预后的临床因素进入生存回归模型，筛选影响和预测房颤消融术后复发的临床指标。

2、在体功能学研究：

我科建立了华东地区首个同种瓣膜库，在从器官移植供体的正常心脏获得同种瓣膜以后剩余的心脏上剪取左心耳组织 400mg 及其 2ml 静脉血，静脉血同上分离后提取全基因组 DNA，并检测相关基因型；左心耳组织 100mg 用于 real-time PCR 检测，100mg 用于 western blot，100mg 用于 ChIP 试验，另外 100mg 经中性甲醛固定后包蜡用于特殊染色。

① 用 real-time PCR 检测左心耳中 TGF-β1 基因表达水平，比较同一多态性位点不同基因

型间 TGF-β1 基因表达差异；② 用 western blot 检测左心耳中 TGF-β1 蛋白表达水平，比较同一多态性位点不同基因型间 TGF-β1 蛋白表达差异；③ 用 Masson 染色检测左心耳组织中间质纤维化程度，计算胶原容积分数（CVF），比较同一多态性位点不同基因型间心房间质纤维化程度的差异。

3、伦理学考虑：

所有研究对象均获知该研究的目的和意义，签署知情同意书后才能收集他（她）们用于行血常规检查的剩余血，然后在原应丢弃的剩余血中提取全基因组 DNA 用于检测分析。调查对象的所有个人信息、疾病史、家族史、基因信息等均严格保密。至于在体功能学研究，我们是在从器官移植供体的正常心脏获得同种瓣膜以后剩余的心脏上剪取的左心耳组织，然后在这部分本应丢弃的组织上进行重要的功能学研究。

伦理委员会审查意见

经本单位伦理委员会审核，此项目符合卫生部《涉及人的生物医学研究伦理审查办法（试行）》及赫尔辛基宣言关于生物学人体试验的相关规定，同意开展研究。

伦理委员会主任委员签字：[illegible]

伦理委员会章：[illegible]　　年　　月　　日
